# Comparison of Length of Hospital Stay for Community-Acquired Infections Due to Enteric Pathogens, Influenza Viruses and Multidrug-Resistant Bacteria: A Cross-Sectional Study in Hungary

**DOI:** 10.3390/ijerph192315935

**Published:** 2022-11-29

**Authors:** Nikolett Orosz, Tünde Tóthné Tóth, Gyöngyi Vargáné Gyuró, Zsoltné Tibor Nábrádi, Klára Hegedűsné Sorosi, Zsuzsa Nagy, Éva Rigó, Ádám Kaposi, Gabriella Gömöri, Cornelia Melinda Adi Santoso, Attila Nagy

**Affiliations:** 1 Department of Hospital Hygiene, University of Debrecen Clinical Centre, 4032 Debrecen, Hungary; 2 Department of Health Informatics, Faculty of Health Sciences, University of Debrecen, 4028 Debrecen, Hungary

**Keywords:** community-acquired infections, gastroenteritis, enteric pathogen, respiratory infections, influenza, multidrug-resistant bacteria, length of hospital stay, mortality, Hungary

## Abstract

Community-acquired infections (CAI) can affect the duration of care and mortality of patients. Therefore, we aimed to investigate these as well as factors influencing the length of hospital stay in patients with CAI due to enteric pathogens, influenza viruses and multidrug-resistant (MDR) bacteria. We obtained data on 531 patients with CAI from the medical databases of a Hungarian university hospital and analyzed their characteristics using a regression model. Patients with MDR bacterial infection had the highest mortality (26.24%) and they stayed significantly longer in the hospital than cases with other CAIs. Our results showed that infection by *Clostridioides difficile* (odds ratio (OR): 6.98, 95% confidence interval (CI): 1.03–47.48; *p* = 0.047), MDR *Escherichia coli* (OR: 7.64, 95% CI: 1.24–47.17; *p* = 0.029), MDR *Klebsiella* spp. (OR: 7.35, 95% CI: 1.15–47.07; *p* = 0.035) and hospitalization in the department of pulmonology (OR: 5.48, 95% CI: 1.38–21.76; *p* = 0.016) and surgery (OR: 4.19, 95% CI: 1.18–14.81; *p* = 0.026) significantly increased, whereas female sex (OR: 0.62, 95% CI: 0.40–0.97; *p* = 0.037) and hospitalization in the department of pediatrics (OR: 0.17, 95% CI: 0.04–0.64; *p* = 0.009) decreased the odds of staying in the hospital for more than 6 days. Our findings provide new information on the epidemiology of CAI and can contribute to the development of public health programs that decrease the burden of infections acquired in the community.

## 1. Introduction

Until the late 20th century, communicable diseases were the leading causes of death and thereby the most significant public health issue worldwide [1]. Although morbidity and mortality due to infections have become less common in the past decades, it is still a major challenge to prevent their spread in healthcare facilities [2,3]. Patients with community-acquired infections (CAIs) can introduce pathogenic microorganisms into the hospital environment and be the source of disease outbreaks [4,5,6]. CAIs are defined as infections that are clinically evident at the time of hospital admission or diagnosed within 48 h of it without any previous encounter with healthcare [2]. Gastroenteritis, respiratory infections, and diseases caused by multidrug-resistant (MDR) bacteria are the most frequently detected CAIs [4,5,7,8,9,10,11,12]. 

Globally, infections diagnosed as gastroenteritis are among the most common diseases and they are the leading causes of morbidity and mortality in children under 5 years of age in developing countries [7,8,13]. Of the pathogenic microorganisms causing gastroenteritis, *Campylobacter* and *Salmonella* species are the most important etiological agents of diarrheal infections, contributing to an estimated 7.5 and 4.2 million disability-adjusted life years worldwide, respectively [8,14,15,16,17,18,19]. In addition to these bacteria species, *Clostridioides difficile* infection (CDI) is also a frequent cause of gastroenteritis [8,20,21]. It is estimated that this pathogen alone contributes to the development of 300,000 diarrheal infections and leads to more than 25,000 deaths in North America each year [20]. Epidemiological studies have demonstrated that the incidence of CDI has increased in both adults and children [21]. It has also been shown that 20–45% of all *C. difficile* cases are due to infection acquired in the community [21]. Besides pathogenic bacteria, viruses, including highly infectious Rotavirus, have also been identified as causes of acute diarrheal diseases [7,8]. It has been reported that Rotaviruses can be the cause of 15% of gastroenteritis in children under 5 years of age in the European Union [22]. According to the same research, 95% of unvaccinated children under 5 years of age are affected by infection with Rotavirus, often resulting in hospitalization due to severe dehydration and diarrhea [22].

Of the community-acquired respiratory infections, illnesses caused by seasonal influenza A and B have the greatest public health importance following coronavirus infections [10,23,24,25,26]. The Global Burden of Disease Study estimated that 9.5 million hospitalizations and 145,000 deaths were attributable to influenza virus infections in 2017 [27]. Patients with influenza virus infection are often hospitalized due to complications associated with the disease [10,26,27]. One of these conditions is community-acquired pneumonia, for which influenza viruses were shown to be the pathogenic agent in 9% of European patients with the illness [23].

In addition to gastroenteritis and respiratory infections, diseases due to MDR bacteria are also among the frequent causes of CAI [28]. They belong to the group of MDR organisms characterized by resistance to one or more classes of antimicrobial agents [12,29]. The spread of MDR bacteria has been shown to be strongly associated with inappropriate or excessive use of antibiotics [28,29]. Although MDR bacteria are typically detected as pathogenic agents of hospital-acquired infections, their transmission in the community can contribute to increased morbidity and mortality in the susceptible population [28,29,30]. According to a recent report on antimicrobial resistance surveillance in the European Union, the number of infections and deaths in this region in 2020 due to community and hospital acquired MDR bacteria was estimated to be 670,000 and 33,000, respectively [29]. Therefore, the community transmission of MDR bacteria is now recognized as a growing public health problem [28,29].

As CAIs caused by enteric pathogens, influenza viruses and MDR bacteria have become more common, the number of complications and deaths related to them has also increased [4,27,28,29,31]. To prevent CAI-associated morbidity and mortality, information is needed on the characteristics of patients hospitalized with these diseases and factors affecting their condition. However, there are only a few studies on the epidemiology of patients with CAI. The investigations available have found that CAIs due to MDR bacteria were the most frequent among the elderly, whereas community-acquired influenza and enteric diseases affected both children and elderly [4,8,10,12,27,29]. In addition, previous studies have suggested that there can be differences in the spread of pathogenic microorganisms causing CAI between the population living in urban and rural areas [32,33,34]. However, data on the share of patients with CAI due to enteric pathogens, influenza viruses and MDR bacteria living in settlements with different characteristics are limited, even in developed countries [32,33,34]. It has also been shown that the length of hospital stay is increased if the patients suffered from CAI caused by enteric pathogens, influenza viruses or MDR bacteria [5,35,36]. In addition, there is an association between the length of hospital stay and mortality among patients with CAI [6,27,37]. However, there is no study that simultaneously investigated which of these diseases can result in the longest hospital stay and the highest mortality among patients with CAI. To identify factors influencing the length of hospital stay for patients with CAI, only a few epidemiological investigations have developed regression models focusing mainly on community-acquired pneumonia [38,39]. According to our knowledge, there are no studies using regression analysis that report which type of CAIs and patient characteristics have the greatest influence on the length of hospital stay.

We are, however, able to complement the existing evidence by analyzing data obtained from a Hungarian university hospital on the characteristics of patients with CAI caused by enteric pathogens, influenza viruses and MDR bacteria by investigating whether there is a difference in the proportion of patients with CAI by the patient’s place of residence and determining which of the diseases can result in the longest hospital stay and the highest mortality. The other aim of our study was to develop a regression model that incorporates data on hundreds of patients with CAI to identify factors that influence the length of hospital stay of patients with those diseases. 

## 2. Materials and Methods

### 2.1. Study Design

A cross-sectional study was conducted covering the University of Debrecen Clinical Centre Nagyerdei Campus (UDCC NC), Hungary from 1 January to 31 December 2020.

### 2.2. Data Sources

To identify patients with CAI, data on laboratory-confirmed infections were retrieved from the Medbacter microbiology information system of the UDCC NC for the period between 1 January and 31 December, 2020. Next, the date of admission for each patient was obtained from the e-MedSolution medical information system of the UDCC NC. An infection was considered to be community-acquired when it was clinically evident at the time of hospital admission or diagnosed within 48 h of hospital admission without any previous encounter with healthcare [3]. Subsequently, the following data were obtained for each patient from the e-MedSolution medical information system: age, sex, place of residence, time of admission and discharge, hospital ward (place of treatment), time of sampling, test material, microbiological result and previous antibiotic use. The patients’ place of residence was categorized as ‘village’, ‘city’ and ‘cities with county status’ according to the classification system of the Hungarian Central Statistical Office [40]. ‘Village’ is defined as a settlement which has its own individual identity separate from other settlements, and its legal status is other than a town [40]. Settlements are categorized as ‘cities’ if they have some central role in the geographical division of labor, and they are typically not agricultural settlements and have the legal status of a city [40]. Cities with county status are settlements designated by the respective legal rule as the seat of the county [40]. The total number of inpatients treated at the UDCC NC in 2020 was obtained from reports of the Department of Medical Documentation of the University of Debrecen. We had ethical approval from the Regional and Institutional Ethics Committee of the University of Debrecen to process patient data (DE RKEB/IKEB: 5677-2021). 

### 2.3. Inclusion and Exclusion Criteria for Selection of Patients with CAI

**Figure 1:**
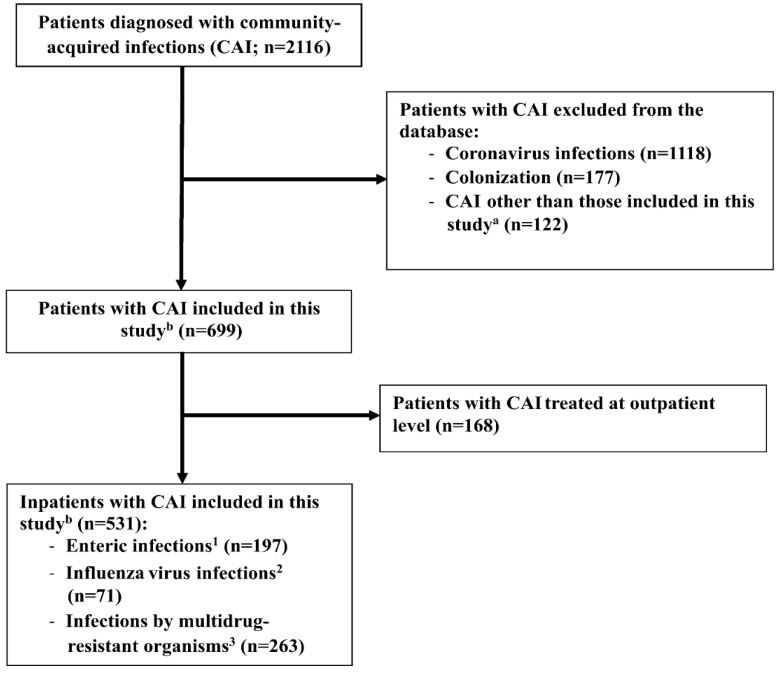
Flowchart of database development. ^a^ Patients with CAI caused by Adenovirus, Hepatitis viruses, Herpes viruses, Respiratory syncitial virus, *Legionella pneumophila*, *Neisseria meningitidis*, *Streptococcus pneumoniae*, *Haemophilus influenzae*, *Mycobacterium tuberculosis*, *Yersinia enterocolitica*, *Shigella sonnei*, Enteropathogen *Escherichia coli*, *Clostridium perfringens*, *Entamoeba hystolitica*, *Giardia lamblia* and HIV virus. ^b^ Patients with CAI caused by *Campylobacter jejuni* ^1^*, Campylobacter coli* ^1^, Rotavirus ^1^, *Salmonella* sp. ^1^, *Clostridioides difficile* ^1^, Influenza A and B viruses ^2^, Methicillin-resistant *Staphylococcus aureus* ^3^, Vancomycin-resistant *Enterococcus* spp. ^3^, Multidrug-resistant (MDR) *Acinetobacter baumannii* ^3^, MDR *Escherichia coli* ^3^, MDR *Enterobacter* spp. ^3^, MDR *Klebsiella* spp. ^3^, MDR *Pseudomonas aeruginosa* ^3^, MDR *Proteus mirabilis* ^3^, MDR *Citrobacter freundii ^3^*, MDR *Morganella morganii* ^3^ and MDR *Stenotrophomonas maltophilia* ^3^.

Our study included inpatients with CAI due to enteric pathogens, influenza viruses and MDR bacteria. However, patients with coronavirus infections, colonization and CAI other than those included in this study were excluded from our research. In addition, outpatients were also omitted from our study (for more details see Figure 1).

### 2.4. Database Development

The number of infections meeting the criteria of CAI was 2116 in the UDCC NC for the period studied. Coronavirus infections were not included in our database because we intended to analyze data on other major causes of CAI. Therefore, patients with coronavirus infections (n = 1118), colonization (n = 177) and CAI other than those included in this study (n = 122, for more details see Figure 1) were excluded from our database. This left 699 patients with CAI infection in our database. Then, patients with CAI treated at outpatient level (n = 168) were also omitted from our database. As a result, 531 patients with CAI remained in our database including enteric (n = 197) and influenza virus (n = 71) infections and diseases caused by MDR bacteria (n = 263). Enteric infections included illnesses caused by *Campylobacter jejuni*, *Campylobacter coli*, *Salmonella* sp., *C. difficile* (CDI) and Rotavirus. Respiratory diseases by Influenza A and B viruses were included in the category of Influenza virus infections. Diseases caused by the following pathogenic microorganisms were categorized as infections by MDR bacteria: Methicillin-resistant *Staphylococcus aureus* (MRSA), MDR *Escherichia coli* (MECO), MDR *Klebsiella* spp., (MKLE), MDR *Pseudomonas aeruginosa* (MPAE), MDR *Acinetobacter baumannii* (MACI), MDR *Enterobacter* spp. (MENB), MDR *Stenotrophomonas maltophilia* (MSTM), other MDR species (*Proteus mirabilis*, *Citrobacter freundii*, *Morganella morganii*, MR Other) and Vancomycin-resistant *Enterococcus* spp. (VRE). The steps of database development are shown in Figure 1.

### 2.5. Data Analysis

To characterize the sample population, the proportion of patients with CAI was calculated by gender, 10-year age groups, previous antibiotic use, place of residence, causes of CAI and outcome of illnesses (see Table 1). The percentage of patients with CAI was also determined by the type of CAI and place of treatment (hospital ward) (see Table 2). To determine the number of patients with CAI per 100,000 inpatients at the UDCC NC in 2020, the number of patients with each type of CAI was divided separately by the total number of inpatients treated at the UDCC NC in 2020 and multiplied by 100,000. Then, the number of days of care was calculated for each patient with CAI. Subsequently, the median length of hospital stay and the corresponding interquartile ranges (IQR) were calculated separately for patients with CAI by enteric pathogens, influenza viruses and MDR bacteria. To determine whether there is a difference between the length of hospital stay of patients with these CAIs, we used the Kruskal–Wallis test with the Bonferroni post-hoc test. To show which patients had the longest hospital stay among the CAI types included in our study, the median number of days of care and the corresponding interquartile ranges (IQR) were calculated separately for each type of CAI. Next, we applied the Chi-square and the Fischer exact test to determine whether there is a difference within each type of CAI in the proportion of patients by the patient’s place of residence (village, city and cities with county status; see the details on settlement categories in Section 2.2). Then, cases with CAI were divided into two groups based on the median length of hospital stay of all patients with CAI. Group 1 and group 2 included patients for whom the duration of hospitalization was less than or equal to the median and more than the median, respectively. The Chi-square test or the Fischer exact test were applied for each type of CAI separately to see whether there is a difference between the proportions of patients whose care lasted fewer and more days than the median hospital stay of all patients with CAI. To identify factors that influence the length of hospital stay for patients with CAI, we developed a multiple binary logistic regression model. The dependent variable in the logistic regression model was the median number of days of care (≤median; median<), and the independent variables were the patient’s sex and age, place of residence, type of CAI, and place of treatment (hospital ward). Logistic regression was performed using the forced entry method. Variables were included in the initial model if there was an association with length of hospital stay in the scientific literature or the univariate analysis showed a statistically significant relationship between them. The final model included gender as a general confounder. Regression analysis included only those types of CAI and hospital wards which had at least 15 and 13 patients, respectively. The model fit was assessed using Nagelkerke R^2^. The results were considered to be statistically significant when *p* < 0.05. Statistical analyses were performed using IBM SPSS version 28.0.1 (IBM Inc, Armonk, New York, NY, USA).

## 3. Results

**Figure 2:**
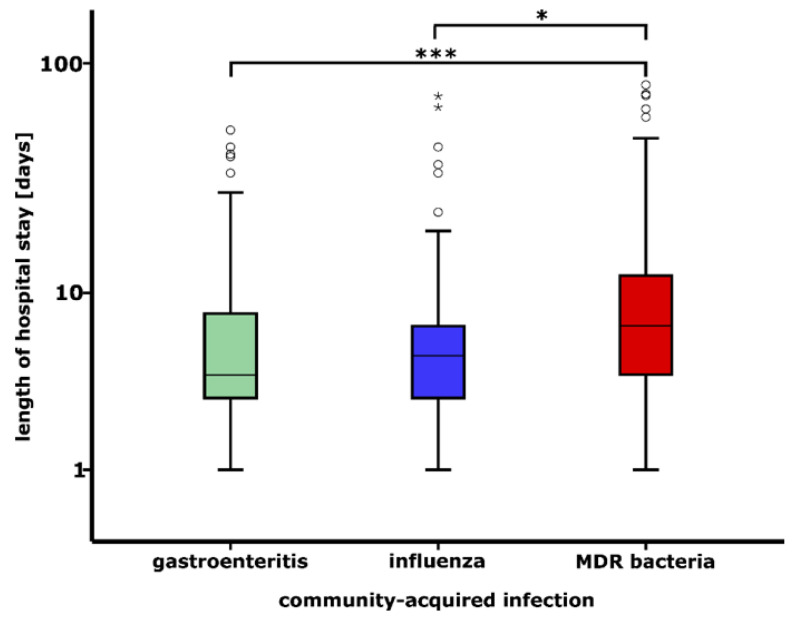
Distribution of length of hospital stay of patients with community-acquired gastroenteritis, influenza and multidrug-resistant infections detected at the University of Debrecen Clinical Centre Nagyerdei Campus in 2020. Median, interquartile ranges and 1.5 times of interquartile ranges as whiskers are shown. Open circles and asterisks indicate outlier values. * *p* < 0.05, *** *p* < 0.001. Gastroenteritis includes patients with community-acquired *Clostridioides difficile, Campylobacter* sp., *Salmonella* sp. and Rotavirus infections. Influenza includes patients with community-acquired Influenza A and B viruses infections. MDR bacteria includes patients with community-acquired Methicillin-resistant *Staphylococcus aureus*, Vancomycin-resistant *Enterococcus* spp., Multidrug-resistant (MDR) *Acinetobacter baumannii*, MDR *Escherichia coli*, MDR *Enterobacter* spp., MDR *Klebsiella* spp., MDR *Pseudomonas aeruginosa*, MDR *Proteus mirabilis*, MDR *Citrobacter freundii*, MDR *Morganella morganii* and MDR *Stenotrophomonas maltophilia* infections.

Table 1 shows that the number of males and females were 256 (48.21%) and 275 (51.79%) among patients with CAI, respectively. It is also demonstrated that the largest proportion of patients with CAI originated from the age groups of 0–9 (32.96%, n = 175), 60–69 (14.31%, n = 76), 70–79 (16.76%, n = 89), and 80–89 (16.57%, n = 88) years (Table 1). The mean age ± SD of all the patients with CAI was 46.68 ± 34.48 years. When taking into account the age of patients with CAI separately by enteric pathogens, influenza viruses and MDR bacteria, the mean age ± SD was found to be 22.79 ± 31.30 years, 29.09 ± 29.58 years and 69.32 ± 20.27 years, respectively. Previous antibiotic use in the past 30 days was recorded in 10 cases (1.88%) (Table 1). In addition, CAIs included in this study were detected more frequently among patients living in cities with county status (46.52%) than among those living in other types of settlement (Table 1). MDR bacteria were found to be to the cause of CAI in 49.53% of the patients included in this study (Table 1). Table 1 also illustrates that 83.24% of patients with CAI recovered from the disease. As shown in Table 1, in-hospital mortality was the highest among patients with CAI due to MDR bacteria (26.24%). Of the enteric pathogens studied, *Salmonella* sp. (n = 62, 31.47%, 86.76 cases/100,000 inpatients) were the most frequent cause of gastroenteritis at the UDCC NC in 2020 (Table 2). The number of patients with Influenza virus infection was 99.35/100,000 inpatients (n = 71, Table 2). It is also shown that among the MDR bacteria MECO (n = 102, 38.78%, 142.73 cases/100,000 inpatients) caused the highest number of infections (Table 2). As shown in Table 2, patients with CAI were detected most frequently at hospital wards of Pediatrics (n = 192, 36.16%) and General medicine A (n = 106, 19.96%) and B (n = 111, 20.90%). Figure 2 depicts that the length of hospital stay of patients with MDR bacterial infection was significantly longer than that of patients with gastroenteritis (*p* < 0.001) and influenza (*p* < 0.05).

**Figure 3:**
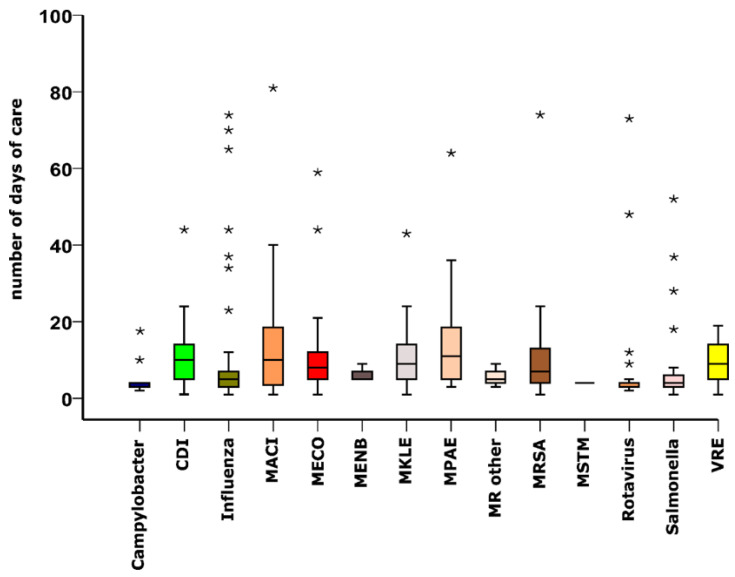
Number of days of care of patients with community-acquired infections detected at the University of Debrecen Clinical Centre Nagyerdei Campus in 2020 by the type of infectious disease. Median, interquartile ranges and 1.5 times of interquartile ranges as whiskers are shown. Asterisks indicate outlier values. Abbreviations: CDI: *Clostridioides difficile*, MRSA: Methicillin-resistant *Staphylococcus aureus*, MECO: Multidrug-resistant *Escherichia coli*, MKLE: Multidrug-resistant *Klebsiella* spp., MPAE: Multidrug-resistant *Pseudomonas aeruginosa*, MACI: Multidrug-resistant *Acinetobacter baumannii*, MENB: Multidrug-resistant *Enterobacter* spp., MSTM: Multidrug-resistant *Stenotrophomonas maltophilia*, MR OTHER: Other Multidrug-resistant organisms (*Proteus mirabilis, Citrobacter freundii, Morganella morganii*), VRE: Vancomycin-resistant *Enterococcus* spp.

**Figure 4:**
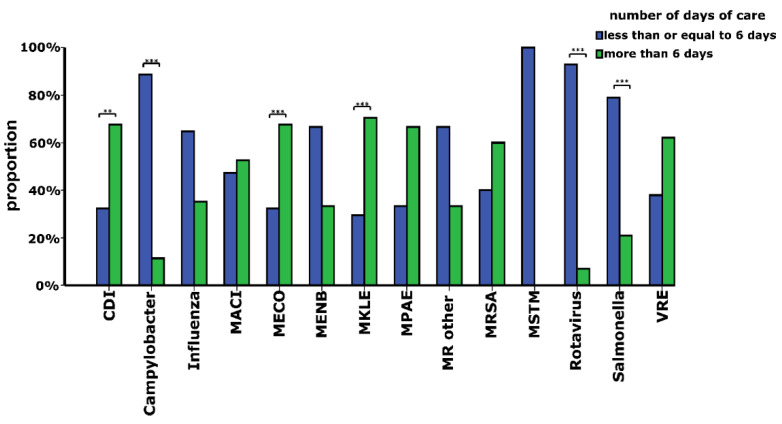
Distribution of patients with community-acquired infections detected at the University of Debrecen Clinical Centre Nagyerdei Campus by the median number of days of care in 2020. Patients with community-acquired infections were divided into two groups based on the median number of days of care (≤6 days versus >6 days of hospitalisation). Abbreviations: CDI: *Clostridioides difficile*, MRSA: Methicillin-resistant *Staphylococcus aureus*, MECO: Multidrug-resistant *Escherichia coli*, MKLE: Multidrug-resistant *Klebsiella* spp., MPAE: Multidrug-resistant *Pseudomonas aeruginosa*, MACI: Multidrug-resistant *Acinetobacter baumannii*, MENB: Multidrug-resistant *Enterobacter* spp., MSTM: Multidrug-resistant *Stenotrophomonas maltophilia*, MR OTHER: Other Multidrug-resistant organisms (*Proteus mirabilis, Citrobacter freundii, Morganella morganii*), VRE: Vancomycin-resistant *Enterococcus* spp. ** *p* < 0.01, *** *p* < 0.001.

**Figure 5:**
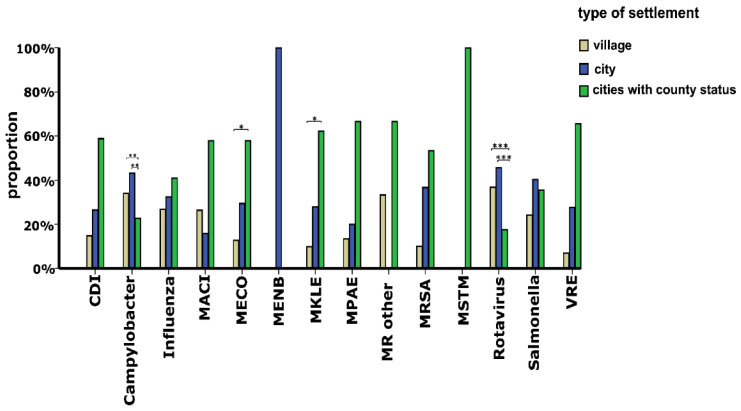
Distribution of patients with community-acquired infections detected at the University of Debrecen Clinical Centre Nagyerdei Campus in 2020 by place of residence. Place of residence was categorised as village, city and cities with county status (see the details on settlement categories in Section 2.2). Abbreviations: CDI: *Clostridioides difficile*, MRSA: Methicillin-resistant *Staphylococcus aureus*, MECO: Multidrug-resistant *Escherichia coli*, MKLE: Multidrug-resistant *Klebsiella* spp., MPAE: Multidrug-resistant *Pseudomonas aeruginosa*, MACI: Multidrug-resistant *Acinetobacter baumannii*, MENB: Multidrug-resistant *Enterobacter* spp., MSTM: Multidrug-resistant *Stenotrophomonas maltophilia*, MR OTHER: Other Multidrug-resistant organisms (*Proteus mirabilis*, *Citrobacter freundii, Morganella morganii*), VRE: Vancomycin-resistant *Enterococcus* spp. * *p* < 0.05, ** *p* < 0.01, *** *p* < 0.001.

Considering the duration of hospital stay of all patients with CAI, the median number of days of care was 6 (IQR: 2–10). Figure 3 depicts that among the CAI types studied, patients with MPAE (median: 11 days, IQR: 2.5–19.5) and Rotavirus infection (median: 3 days, IQR: 2.5–3.5) had the longest and shortest hospital stay, respectively. Figure 4 illustrates that the proportions of patients with CDI (*p* < 0.01), MECO (*p* < 0.001) and MKLE (*p* < 0.001) infections hospitalized for more than 6 days were significantly larger than those of cases treated for 6 or less days. In contrast, compared with the percentage of patients with *Campylobacter* (*p* < 0.001), *Salmonella* (*p* < 0.001) and Rotavirus (*p* < 0.001) infections treated for more than 6 days, the proportions of cases with those diseases hospitalized for 6 or less days were significantly larger (Figure 4). Figure 5 shows that compared with cases with *Campylobacter* and Rotavirus infection living in cities with county status, significantly larger proportions of patients with those diseases were from villages (*Campylobacter*: *p* < 0.01, Rotavirus: *p* < 0.001) and cities (*Campylobacter*: *p* < 0.01, Rotavirus: *p* < 0.001). It is also demonstrated that significantly larger percentages of patients with MECO and MKLE (*p* < 0.05) infections were from cities with county status than from villages and cities (Figure 5). Multiple binary logistic regression analysis showed that CDI (OR: 6.98, 95% CI: 1.03–47.48; *p* = 0.047), MECO (OR: 7.64, 95% CI: 1.24–47.17; *p* = 0.029) and MKLE (OR: 7.35, 95% CI: 1.15–47.07; *p* = 0.035) infections were independent risk factors for hospital stays longer than 6 days (Table 3). It is also demonstrated that hospitalization at departments of pulmonology (OR: 5.48, 95% CI: 1.38–21.76; *p* = 0.016) and surgery (OR: 4.19, 95% CI: 1.18–14.81; *p* = 0.026) increased the odds of hospital stays longer than 6 days (Table 3). On the other hand, Table 3 shows that female sex (OR: 0.62, 95% CI: 0.40–0.97; *p* = 0.037) and hospitalization at departments of pediatrics (OR: 0.17, 95% CI: 0.04–0.64; *p* = 0.009) decreased the odds of hospital stays longer than 6 days. The Nagelkerke R^2^ for the model was found to be 0.435.

**Table 1:** Characteristics of patients with community-acquired infections.

Variables	N *	%
**Sex**		
Female	275	51.79
Male	256	48.21
**Age groups (years)**		
0–9	175	32.96
10–19	21	3.95
20–29	6	1.13
30–39	8	1.51
40–49	12	2.26
50–59	37	6.97
60–69	76	14.31
70–79	89	16.76
80–89	88	16.57
90 and older	19	3.58
Previous antibiotic use **	10	1.88 ***
**Place of residence ^+^**		
village	107	20.15
city	177	33.33
cities with county status	247	46.52
**Causes of CAI**		
Enteric pathogens ^a^	197	37.1
Respiratory pathogens ^b^	71	13.37
Multidrug-resistant bacteria ^c^	263	49.53
**Outcome of illness**		
Enteric pathogens		
Recovered	185	93.91
In-hospital mortality	12	6.09
Respiratory pathogens		
Recovered	63	88.73
In-hospital mortality	8	11.27
Multidrug-resistant bacteria		
Recovered	194	73.76
In-hospital mortality	69	26.24
Total		
Recovered	442	83.24
In-hospital mortality	89	16.76

* Total number of cases was 531. ** Previous antibiotic use in the past 30 days. *** The proportion of those CAI patients who used antibiotics in the 30 days prior to their infection. ^+^ See the details on settlement categories in Section 2.2. ^a^ *Campylobacter jejuni*, *Campylobacter coli*, Rotavirus, *Salmonella* sp., *Clostridioides difficile*; ^b^ Influenza A and B viruses; ^c^ Methicillin-resistant *Staphylococcus aureus*, Vancomycin-resistant *Enterococcus* spp., Multidrug-resistant (MDR) *Acinetobacter baumannii*, MDR *Escherichia coli*, MDR *Enterobacter* spp., MDR *Klebsiella* spp., MDR *Pseudomonas aeruginosa*, MDR *Proteus mirabilis*, MDR *Citrobacter freundii*, MDR *Morganella morganii* and MDR *Stenotrophomonas maltophilia*.

**Table 2:** Distribution of patients by type community-acquired infections, hospital ward and number of cases/100,000 inpatients.

Variables	N *	%	Number of Cases/100,000 Inpatients
***Cause of community-acquired infections***			
**Enteric pathogens**			
*Campylobacter*	44	22.34	61.57
Rotavirus	57	28.93	79.76
*Salmonella*	62	31.47	86.76
*Clostridioides difficile*	34	17.26	47.58
**Respiratory pathogens**			
Influenza	71	100	99.35
**Multidrug-resistant bacteria**			
MACI	19	7.22	26.59
MECO	102	38.78	142.73
MENB	3	1.14	4.2
MKLE	61	23.2	85.36
MPAE	15	5.7	20.99
MR OTHER	3	1.14	4.2
MRSA	30	11.41	41.98
MSTM	1	0.38	1.4
VRE	29	11.03	40.58
***Hospital wards***			
Cardiology	2	0.38	
Dermatology	7	1.32	
General medicine (A) **	106	19.96	
General medicine (B) **	111	20.9	
General medicine (C) **	29	5.46	
Gynaecology	6	1.13	
Neurosurgery	1	0.19	
Neurology	3	0.56	
Otolaryngology	1	0.19	
Orthopaedics	3	0.56	
Ophthalmology	1	0.19	
Pediatrics	192	36.16	
Pulmonology	23	4.33	
Surgery	33	6.22	
Urology	13	2.45	

* Total number of cases was 531. ** General Medicine ward A includes the following specialties: general cardiology, diabetology, endocrinology, nephrology and obesitology. General Medicine ward B includes the following specialties: hematology and rare diseases. General Medicine ward C includes the following specialties: angiology, immunology and geriatric medicine. Abbreviations: MACI: Multidrug-resistant *Acinetobacter baumannii*, MECO: Multidrug-resistant *Escherichia coli*, MENB: Multidrug-resistant *Enterobacter* spp., MKLE: Multidrug-resistant *Klebsiella* spp., MPAE: Multidrug-resistant *Pseudomonas aeruginosa*, MR OTHER: Other multidrug-resistant organisms (*Proteus mirabilis, Citrobacter freundii, Morganella morganii*), MRSA: Methicillin-resistant *Staphylococcus aureus*, MSTM: Multidrug-resistant *Stenotrophomonas maltophilia*, VRE: Vancomycin-resistant *Enterococcus* spp.

**Table 3:** Significant influencing factors of hospital stays longer than 6 days for patients with community-acquired infections based on single and multiple logistic regression model.

Single Logistic Regression Model							Multiple Logistic Regression Model					
Variable	β	S.E.	uOR	95% CI for uOR		*p*-Values	β	S.E.	aOR	95% CI for aOR		*p*-Values
				Lower	Upper					Lower	Upper	
Female ^a^	−0.07	0.18	0.93	0.66	1.31	0.689	-0.48	0.23	0.62	0.40	0.97	0.037
Microbiological result ^b^												
CDI	0.99	0.38	2.70	1.29	5.66	0.009	1.94	0.98	6.98	1.03	47.48	0.047
MECO	1.15	0.23	3.16	2.00	4.99	<0.001	2.03	0.93	7.64	1.24	47.17	0.029
MKLE	1.20	0.30	3.31	1.85	5.91	<0.001	1.99	0.95	7.35	1.15	47.07	0.035
Hospital wards ^c^												
Pediatrics	−2.91	0.27	0.06	0.03	0.09	<0.001	-1.79	0.69	0.17	0.04	0.64	0.009
Pulmonology	1.82	0.56	6.17	2.07	18.39	0.001	1.70	0.70	5.48	1.38	21.76	0.016
Surgery	1.75	0.50	5.77	2.15	15.49	<0.001	1.43	0.65	4.19	1.18	14.81	0.026

Abbreviations: S.E.: standard error, uOR: unadjusted odds ratio, aOR: adjusted odds ratio, CI: Confidence interval, CDI: *Clostridioides difficile*, MECO: Multidrug-resistant *Escherichia coli*, MKLE: Multidrug-resistant *Klebsiella* spp. Values of *p* < 0.05 were considered statistically significant. ^a^ reference: male; the final model included gender as a general confounder, ^b^ reference: patients with other types of community-acquired infections, ^c^ reference: patients with community-acquired infections treated at other hospital wards.

## 4. Discussion

Community-acquired infectious diseases can increase the risk of complications, thereby often resulting in longer hospitals stays for patients with these illnesses. In addition, mortality has been shown to be increased among patients with CAI. However, it has not been previously known which of type of CAI can result in the longest hospital stay and the highest mortality. Factors influencing the length of hospital stay of patients with CAI due to enteric pathogens, influenza viruses and MDR bacteria have also not been investigated. To fill this knowledge gap, we obtained data on 531 patients with CAI from the medical databases of UDCC NC then used them to analyze patient characteristics and develop a regression model. Our results showed that compared with cases with *Campylobacter* and Rotavirus infection living in cities with county status, significantly larger proportions of patients with those diseases were from villages and cities. In addition, significantly larger percentages of patients with MECO and MKLE infections were from cities with county status than from villages and cities. We also found that patients with MDR bacterial infection had the highest mortality (26.24%) and they stayed significantly longer in hospitals than cases with other types of CAIs. Of the CAIs by MDR bacteria, infections due to MECO were shown to be the most frequent (n = 102, 38.78%, 142.73 cases/100,000 inpatients). Considering all patients with CAI, the median length of hospital stay was found to be 6 days. Our study showed that among the patients with CAI, those with MPAE and Rotavirus infections had the longest (median: 11 days) and the shortest (median: 3 days) hospital stay, respectively. In addition, it was also demonstrated that hospitalization for more than 6 days was significantly more frequent among patients with CDI, and infections by MECO and MKLE. Using multiple logistic regression, we have identified five and two factors that significantly increased (CAI by CDI, MECO and MKLE, hospitalization at department of pulmonology and surgery) and decreased (female sex, hospitalization at department of pediatrics) the odds of staying in the hospital for more than 6 days, respectively. 

Prior investigations have reported that the spread of pathogenic microorganisms causing CAI can be facilitated by the urban environment including densely built city centers, crowded public transportation and close contact with companion animals such as dogs and cats [32]. Therefore, it is assumed that people’s place of residence can influence the type of pathogenic microorganisms they are exposed to on a daily basis [32]. This might contribute to differences in the share of patients with CAI due to enteric pathogens, influenza viruses and MDR bacteria in villages, cities, and cities with county status [32,33,34]. Our results support this assumption for CAI due to *Campylobacter*, Rotavirus, MECO and MKLE; however, further research is required to determine the role of environment at a settlement in the spread of CAIs.

Previous epidemiological studies have shown that CAI due to MDR bacteria can result in long hospital stays and high mortality of patients [41]. Our results confirmed these findings, demonstrating that mortality from MDR bacterial infection was more than twice and four times higher than that of from influenza virus infection and gastroenteritis, respectively. In addition, the median number of days of care of patients with MDR bacterial infection was 2.0 and 1.6 times higher than for cases with gastroenteritis and influenza, respectively. The results obtained could be due the differences in the age of patients since advanced age has been identified as one of the major risk factors for mortality from MDR infections [42,43]. This possible explanation is also supported by the results of our investigation, as the mean age of patients with MDR bacterial infection was more than twice that of the cases with gastroenteritis and influenza. Therefore, among CAI, MDR infections should be considered as a public health priority in populations vulnerable to them.

To determine how the median length of hospital stays of patients with MPAE infection in our study relates to those described in previous research, we compared our results with the existing evidence. According to two recent publications from the United States and Australia, the median length of hospital stay of patients with MPAE infections was 8 days which is in accordance with our results (median: 11 days) [36,44]. The reason for the long hospital stays of these patients can be related to complications including pneumonia and bacteremia that often develop as a result of MPAE infection [44,45,46]. Clinical studies have shown that delayed identification of MPAE infection associated pneumonia can increase not only the duration of care, but also its treatment cost and mortality [44,45]. Therefore, the early detection of the disease followed by therapy with adequate antibiotics is essential to decrease the burden related to CAI by MPAE [44]. 

Similar to CAI due to MPAE, infections with *C. difficile*, MECO and MKLE are often associated with severe diseases such as diarrhoea, urinary tract infections, and blood stream infections [47,48,49,50]. Previous studies have shown that a large proportion of patients with these illnesses have pre-existing risk factors including smoking, high blood glucose levels, high body mass index, previous antibiotic use and advanced age [42,47]. Although, data on risk factors for cases with *C. difficile*, MECO and MKLE infections were not available in our study, we hypothesize that they may have contributed to the higher proportion of hospitalization for more than 6 days among these patients. Our results indicate that further research is needed to determine the effects of pre-existing risk factors on the length of hospitalization for patients with community-acquired *C. difficile*, MECO and MKLE infections.

The results of our regression model suggest that the length of hospital stay can depend on the health status of the patients. Compared with patients without comorbidities, the duration of recovery from community-acquired infections is longer for those with immunosuppression, cancer and chronic respiratory diseases [36,51]. Previous studies have shown that differences in health and lifestyle among men and women in combination with CAI can lead to longer hospital stays [52,53]. In addition, it has been reported that in males, infections are at a more advanced stage when they are admitted to hospital, further increasing the length of hospitalization [54]. This is consistent with the results of our regression analysis, which showed that women are 0.38 times less likely than men to stay in the hospital for more than 6 days. Following the same logic, it is assumed that *C. difficile*, MECO and MKLE infections independently increase the odds of hospital stays longer than 6 days because patients with these infections often admitted with poor health and comorbidities [41,47,48,49,50]. Patients admitted with comorbidities are not evenly distributed between hospital wards, those with cancer and chronic respiratory diseases can be present in a much larger proportions among adults in pulmonology and surgery units than among children in the pediatric ward [55,56]. Therefore, patients with CAI treated in pulmonology and surgery wards have 5.48 and 4.19 times higher odds, respectively, of being hospitalized for more than 6 days compared with CAI patients treated in other units.

The strengths and limitations of this study should be considered. This is the first study that compares the characteristics of patients with different types of CAI in the same university hospital. Another strength of the investigation is that besides adults, it also includes children with CAI. Furthermore, mortality and length of hospital stay due to different types of CAIs was compared. We also demonstrated that infection with *C. difficile*, MECO and MKLE often leads to hospitalization for more than 6 days in a large proportion of patients. Our research also has several limitations. First, data on patients with CAI were obtained only from one hospital in Hungary; this can make it difficult to generalize our results to patients treated in other hospitals. Second, our study included only those patients with CAI who were admitted to the hospital in 2020. Third, outpatients with CAI were excluded from our investigation. Fourth, there were types of MDR bacteria which were detected only in a few cases; this can increase the uncertainty of the results related to them.

## 5. Conclusions

In summary, our research showed that enteric pathogens, influenza viruses and MDR bacteria are common causes of CAIs leading to hospitalization and mortality. Infection with MECO, MKLE and *C. difficile* led to hospitalization for more than 6 days in a large proportion of patients with those CAIs. Considering in-hospital mortality and the duration of care, CAIs caused by MDR bacteria have been found to be of the greatest clinical importance. The results of our regression analysis suggest that the length of hospital stay of patients with CAI can depend on the health status and the comorbidities of the infected person. However, further studies are required to determine how patient characteristics influence the duration of hospital stay and outcome of the illness in CAI.

Our findings provide new information on patients with CAI which can help to better understand the epidemiology these diseases. Together with the evidence from previous studies, our results can contribute to the development hospital antibiotic stewardship and community-based programs that are urgently needed to degrease the public health burden of infections acquired in the community. Further studies are needed to characterize cases with CAI, especially those caused by MDR bacteria, to understand which factors increase the length of hospital stay and risk of death in patients.

## Data Availability

The data underlying this article cannot be shared publicly due to the privacy of patients involved and legal reasons. The anonymized and aggregated data will be available upon request to interested and qualified researchers. Data requests should be sent to the corresponding author.
